# Matrix exopolysaccharides; the sticky side of biofilm formation

**DOI:** 10.1093/femsle/fnx120

**Published:** 2017-06-12

**Authors:** Eve Maunders, Martin Welch

**Affiliations:** Department of Biochemistry, University of Cambridge, Cambridge CB2 1QW, UK

**Keywords:** biofilm: exopolysaccharides, *Pseudomonas aeruginosa*, matrix polysaccharide, cystic fibrosis, c-di-GMP

## Abstract

The Gram-negative pathogen *Pseudomonas aeruginosa* is found ubiquitously within the environment and is recognised as an opportunistic human pathogen that commonly infects burn wounds and immunocompromised individuals, or patients suffering from the autosomal recessive disorder cystic fibrosis (CF). During chronic infection, *P. aeruginosa* is thought to form structured aggregates known as biofilms characterised by a self-produced matrix which encases the bacteria, protecting them from antimicrobial attack and the host immune response. In many cases, antibiotics are ineffective at eradicating *P. aeruginosa* from chronically infected CF airways. Cyclic-di-GMP has been identified as a key regulator of biofilm formation; however, the way in which its effector proteins elicit a change in biofilm formation remains unclear. Identifying regulators of biofilm formation is a key theme of current research and understanding the factors that activate biofilm formation may help to expose potential new drug targets that slow the onset of chronic infection. This minireview outlines the contribution made by exopolysaccharides to biofilm formation, and describes the current understanding of biofilm regulation in *P. aeruginosa* with a particular focus on CF airway-associated infections.

## Teaser

Here, we review our understanding of what controls production of the sticky ‘glue’ that holds bacterial communities together.

## INTRODUCTION

*Pseudomonas aeruginosa* is an opportunistic human pathogen that is particularly dreaded by the clinical community. This Gram-negative bacterium has been isolated from burn wounds, and from the respiratory, gastrointestinal and urinary tracts, and wreaks havoc within clinical environments due to its ability to grow on medical equipment such as catheters, stents and ventilators. This creates a high risk of transmission between patients and has afforded *P. aeruginosa* the dubious accolade of most common nosocomial infection (Pereira *et al.*^2014^). The ability of *P. aeruginosa* to adapt and persist in a wide variety of environments can be attributed to its large genome (6.3 Mbp in size) and flexible metabolism. Whilst most of the time it exists as a relatively harmless microorganism, an infection in immunocompromised individuals or patients receiving immunosuppressive drugs can rapidly become severe enough to result in death of the patient. Treatment options for the infection are also often limited as *P. aeruginosa* naturally exhibits a high intrinsic resistance to many antimicrobial agents, making it a huge health and economic burden (Taylor, Yeung and Hancock 2014).

*Pseudomonas aeruginosa* is currently the leading cause of morbidity and mortality in immunocompromised patients with, for example, ventilator-associated pneumonia or cystic fibrosis (CF), because it causes irreversible damage to the lung tissue and accelerated lung failure (McCarthy *et al.*^2014^; Raineri *et al.*^2014^). CF is the most prevalent autosomal recessive disorder amongst the Caucasian population (McCarthy *et al.*^2014^) and is caused by mutations in the *CFTR* gene, which encodes a chloride channel essential for maintaining periciliary liquid volume and efficient mucociliary clearance within the lung. As a result, mucus accumulates in the airways and inhaled microbes are not cleared effectively, leading to recurrent infection by pathogens. It is this recurrent infection which aggravates the airways causing chronic inflammation and damage to the lung tissue. The lungs become severely scarred as a result and eventually, function can only be recovered through radical procedures such as transplantation. Loss of lung function due to bacterial infection is therefore one of the most significant causes of deterioration in CF patients (Ratjen and Döring 2003; McCarthy *et al.*^2014^; Trinh *et al.*^2015^).

*Pseudomonas aeruginosa* is well adapted to a microaerobic lifestyle and thrives in the low-oxygen conditions of the CF lung (Ratjen and Döring 2003). In principle, infections can be divided into two broad types: acute infections, which are associated with highly virulent, free-swimming (planktonic) cells that are extremely invasive and cause substantial tissue damage, and chronic infections, which are commonly associated with a sessile mode of growth (biofilms). Biofilms are surface-associated bacterial communities embedded within a self-made, extracellular polymeric matrix. Unlike the situation in planktonic cells, the production of virulence factors and the motility apparatus is downregulated, although this reserved lifestyle still elicits an immune response due to the high titres of bacteria accumulating in the airways. In CF patients, it is the resulting unrelenting inflammatory response that indirectly causes most damage to the host tissue (Alhede *et al.*^2009^).

Current treatment regimens involve aggressive use of high dose antibiotics and combination therapy. Diversity within the *P. aeruginosa* population creates a reservoir of antibiotic-resistant mutants, so although treatment often temporarily improves lung function, it is rarely effective in eradicating chronic infections (Mowat *et al.*^2011^; Milla *et al.*^2014^). Furthermore, antibiotic treatment could potentially worsen the condition by selecting for ‘persister’ cells within the biofilm which readily recolonise the CF airways following treatment leading to recurrent infection (Miller *et al.*^2014^).

So far it remains unclear exactly what triggers the planktonic to biofilm growth transition. This is an important problem because identifying global biofilm regulators may offer therapeutic opportunities for the treatment of chronic infection, and this has become a major focus of current research. This minireview outlines our current understanding of *P. aeruginosa* biofilm development, how biofilms are formed in the CF airways and some of the known pathways involved in biofilm regulation.

## BIOFILM DEVELOPMENT AND THE MATRIX STRUCTURE

A number of technological advances have catalysed insight into the development of bacterial biofilms. In particular, the development of flow cell biofilm chambers (which can be monitored using laser scanning confocal microscopy) has revealed the temporal changes which accompany biofilm formation *in vitro*. These studies have revealed that biofilm formation follows a sequential process of attachment, growth, maturation and dispersal, and high-throughput screening has aided in the discovery of many of the fundamental genetic factors required for biofilm formation. Moreover, details of the physical and chemical communication systems and ‘social interactions’ within the biofilm are now being unearthed (Klausen *et al.*^2003^; Müsken *et al.*^2010^). Detailed biochemical and genetic analyses are revealing how the self-produced extracellular matrix secreted by the biofilm community is a complex structure composed of exopolysaccharides, DNA and protein, and that this matrix plays a key role by forming an interactive scaffold to support the biofilm's architecture and promote cell–cell communication (Liao, Schurr and Sauer 2013).

The transition from the planktonic growth mode to biofilm formation involves several distinct steps, and progression through these steps is arrested in the absence of certain genes. In 1998, O’Toole and Kolter published the first global genetic screen aimed at identifying *P. aeruginosa* mutants that are defective in biofilm formation, an approach that highlighted the importance of Surface Attachment Defective (*sad*) genes. Several *sad* genes are required for flagellar synthesis, and functional disruption of these genes blocks biofilm formation at a very early stage; surface attachment. This is because the flagellum is involved in overcoming surface repulsion at the liquid–surface interface, thereby enabling temporary surface attachment during the first stage of biofilm formation. Temporary surface attachment is followed by exploration of the surface via type IV pili-dependent twitching motility (Müsken *et al.*^2010^). This way, the surface-associated bacteria come together (presumably through some form of chemoattraction) to form microcolonies. However, in the CF airways, most (ca. 95%) of the *P. aeruginosa* are not actually found attached to the airway epithelial surface but instead live as aggregates suspended within the mucus layer (McCarthy *et al.*^2014^). The bacteria divide in these suspended clusters, eventually forming ‘floating’ microcolonies.

**Figure 1.**
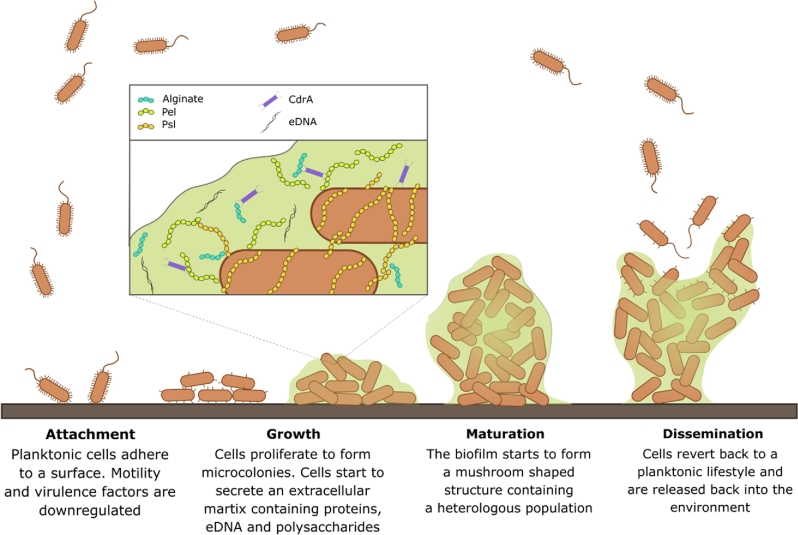
Biofilm formation begins when highly motile planktonic cells adhere to a surface. The cells undergo physical changes that involve downregulation of motility apparatus and virulence factors whilst upregulating the production of an exopolysaccharide-rich extracellular matrix. The community matures into dense mushroom-shaped structures. In response to inducing conditions, cells become motile once again and disseminate to colonise new ecological niches.

As outlined above, microcolony formation is accompanied by a reduction in motility and virulence gene expression (Klausen *et al.*^2003^). Concomitant with this, the microcolonies grow in size to form larger cellular aggregates (Landry *et al.*^2005^). As this happens, large volumes of characteristic extracellular matrix are secreted. The majority of the matrix is made from a viscous mixture of extracellular polysaccharides, principally Psl, Pel and alginate. Proteins such as CdrA act as ‘spars’ between matrix components, adding strength to the structural scaffold (Fig. 1), and extracellular DNA (eDNA) is present in the matrix, providing further structural rigidity. In continuous flow biofilms, the colonies eventually develop into protruding mushroom-shaped structures. However, when conditions require it, the cells on the periphery of the structure are capable of reverting to a planktonic phenotype where they begin to produce enzymes which cleave the matrix components, thereby allowing cells to disseminate into the surrounding environment and colonise new niches (O’Toole and Kolter 1998; Fazli *et al.*^2014^).

Of the three exopolysaccharides (Psl, Pel and alginate) secreted by *Pseudomonas aeruginosa*, alginate is the major polysaccharide produced in mucoid CF isolates. Alginate is a capsule-like exopolysaccharide composed of α-D-mannuronic acid and gluronic acid, producing a negatively charged copolymer. Its secretion gives the colony a mucoid phenotype and the conversion to an alginate overproduction phenotype is essentially pathognomic of CF (Pulcrano *et al.*^2012^).

**Figure 2.**
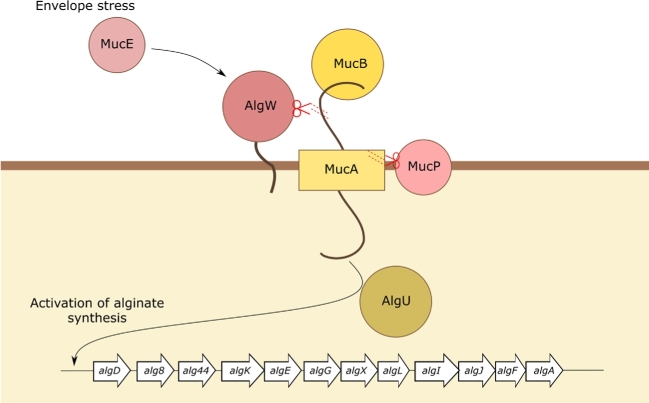
Synthesis and secretion of the exopolysaccharide alginate is regulated through the alternative sigma factor, AlgU. Typically, AlgU is sequestered by the inner membrane-bound protein, MucA. The AlgU-MucA complex is stabilised by MucB. MucE responds to envelope stress by activating the proteolytic activity of AlgW. AlgW and MucP sequentially cleave MucA causing it to release AlgU which is then free to activate transcription of the *algD* operon.

This phenotype arises through transcriptional activation, by AlgU, of the 12 genes required for alginate biosynthesis encoded on the *algA*-*algD* operon. Typically, a membrane-embedded anti-sigma factor, MucA, binds and sequesters the sigma factor AlgU; an interaction stabilised by MucB (Fig. 2). AlgW promotes alginate synthesis by disrupting MucA sequestration of AlgU. Analogous to the well-characterised extracytoplasmic function sigma factor class (σ^E^), of the envelope stress response system in *Escherichia coli*, AlgW is activated by the cell wall inhibitor D-cycloserine and MucE which respond to envelope stresses such as inhibition of peptidoglycan synthesis, outer membrane perturbation and changes in the expression of outer membrane proteins (Damron, Qiu and Yu 2009; Wood and Ohman 2015). The PDZ domain of AlgW inhibits its own protease activity until binding by MucE. This activates AlgW and promotes the cleavage of MucA which in turn encourages further cleavage of MucA by a second protease, MucP. Inactivation of MucA by AlgW, or through *mucA* mutation, prevents sequestration of AlgU, and therefore, increases the concentrations of ‘free’ AlgU and consequently, the overexpression and secretion of alginate (Qui *et al.*^2007^).

In contrast, Psl and Pel polysaccharides monopolise the matrix in non-mucoid strains, such as environmental and domestic laboratory strains. Pel is a glucose-rich polysaccharide with a particular role in pellicle formation when biofilms grow at a liquid–air interface. A pellicle is a ‘floating biofilm’ which provides the colony access to high concentrations of oxygen and nutrients and can be seen on the surface of standing cultures. The *pel* operon consists of seven genes that encode proteins required for polysaccharide synthesis and transport out of the cell. The overexpression of Pel results in the formation of a thick pellicle in liquid culture and causes plate grown colonies to develop a characteristic wrinkled surface (Friedman and Kolter 2004a).

Biosynthesis of the second non-mucoid polysaccharide, Psl, is encoded by a 15-gene operon which coordinates the construction and secretion of the repeating (*D*-mannose—*D*-glucose—*L*-rhamnose) unit that make up the Psl pentasaccharide (Friedman and Kolter 2004b). Psl, alongside Pel, acts as a scaffold to tether neighbouring cells together and is known to be important in attachment of bacterial cells to mucin and airway epithelial cells in the CF lung. Psl forms fibrous structures that are arranged in a helical pattern around *P. aeruginosa* cells creating a mesh-like structure to which neighbouring cells can bind. Type VI pili-mediated twitching may be key to the formation of Psl patterning as Psl is released during migration of the cells. Psl tracks have been identified behind migrating cells and are thought to provide a guided trail for subsequent bacteria that explore the area, contributing to the aggregation of microcolonies (Wang *et al.*2015a). Overexpression of Psl results in cell aggregation when grown in liquid culture, demonstrating its role in cell–cell interactions, and causes increased adhesion of cells to microtitre wells during *in vivo* biofilm plate assays, verifying its role in cell–surface interactions. Given its implications in the attachment, maintenance and maturation of biofilm structure, downregulation of *psl* results in sparse and flimsy biofilms (Ma *et al.*^2006^; Borlee *et al.*^2010^).

As chronic infection develops in the CF airways, numerous new growth morphologies appear. Mucoid cells, overproducing alginate, frequent the lungs as well as small colony variants which exhibit an overproduction of exopolysaccharides. Both morphologies form robust biofilms and contribute to the number of persister cells within the lung (Malone 2015).

eDNA has been found to co-localise with Psl, forming thick rope-like structures distributed around cell aggregates, revealing a physical interaction between the two components (Wang *et al.*2015b). The release of eDNA adjusts the hydrophobicity of the bacterial cell surface enabling hydrophobic interactions to form with the substratum or neighbouring cells. After assisting adhesion, eDNA also plays a role in biofilm expansion by facilitating the alignment and trafficking of cells to the leading edge of the biofilm (Wilton *et al.*^2016^). It has been noted that the Psl-DNA fibres are often concentrated around dead cells suggesting that the eDNA is derived from lysed cells; however, the eDNA found within the biofilm is almost entirely derived from leukocytes that would have been recruited to the infection but lysed due to the release of bacterial virulence factors. This suggests that *P. aeruginosa* can also use non-self eDNA to form a strong skeleton to which its community can adhere to and grow. The presence of Psl might be important in protecting the bacteria from the cation chelating properties of eDNA which promote cell lysis (Wang *et al.*2015b; Wilton *et al.*^2016^).

The protein CdrA was first identified through a screen for genes transcriptionally induced by c-di-GMP. Here, its function as a protein component of the biofilm matrix was determined and it was predicted to be a rod-shaped adhesin that acts as a stabilising crossbar between matrix components to increase the structural integrity of the biofilm. *CdrA* is found within a two-gene operon with *cdrB*, forming a two-partner secretion system consisting of a secreted adhesin (CdrA) and its transporter (CdrB). Overexpression of *cdrA* leads to cell aggregation in liquid culture which has been attributed to CdrA binding to Psl exopolysaccharides. *cdrA* mutants form weak biofilms in which Psl is no longer tightly associated with the cells suggesting that CdrA cross-links Psl polysaccharides or tethers Psl to the cell membranes to increase stability within the matrix and promote cell aggregation. Strains defective in Psl synthesis also exhibit reduced cell-associated CdrA levels (Borlee *et al.*^2010^).

Tolerance to antimicrobial drugs is increased up to 1000-fold in biofilm communities compared with free-swimming cells. The transcriptome is drastically altered during biofilm growth and it is thought that protective proteins such as efflux pumps, slower metabolism and antibiotic deactivating enzymes all contribute to the enhanced antibiotic resistance (Liao, Schurr and Sauer 2013; Xu *et al.*^2015^). In addition, the matrix itself is thick with muculent polysaccharides, which provide a physical barrier to prevent the penetration of antimicrobials allowing time for the bacteria to produce protective enzymes (Baker *et al.*^2015^). *Pseudomonas aeruginosa* must also remain inconspicuous within the proinflammatory environment of the CF lung, where the airways are often teeming with host immune cells. Neutrophils and macrophages are recruited to the infection site where they attempt to engulf and kill the invading bacteria by releasing reactive oxygen species (ROS) and digestive enzymes. Several studies have found that surface-bound exopolysaccharides decreased the ability of neutrophils and macrophages to phagocytose the bacteria by blocking the accessibility of bacterial ligands and preventing their recognition (Malone *et al.*^2010^; Mishra *et al.*^2012^).

## REGULATION OF BIOFILM FORMATION

Biofilm formation is a highly regulated process which is finely tuned by a cascade of signals and interconnecting regulatory networks. The composition of the extracellular environment is most likely to be the initial catalyst for progression into a biofilm lifestyle (O’Toole and Kolter 1998) although many of the downstream effectors remain elusive.

Bis-(3’-5’)-cyclic dimeric guanosine monophosphate (c-di-GMP) is a signalling molecule produced by bacteria to control a whole consort of biological processes. At high intracellular concentrations, c-di-GMP is the key regulator in activating biofilm growth. Intracellular pools of c-di-GMP are modulated by diguanylate cyclases (DGC), which synthesise the molecule, and c-di-GMP phosphodiesterases (PDE), which catabolise it. DGCs are easy to spot because they contain a conserved sequence motif (GGDEF), whereas PDEs contain either an EAL motif or an HD-GYP motif. However, it is very difficult to predict *effectors* of c-di-GMP, and the signalling molecule seems to affect a very diverse range of proteins (Römling, Galperin and Gomelsky 2013).

**Figure 3.**
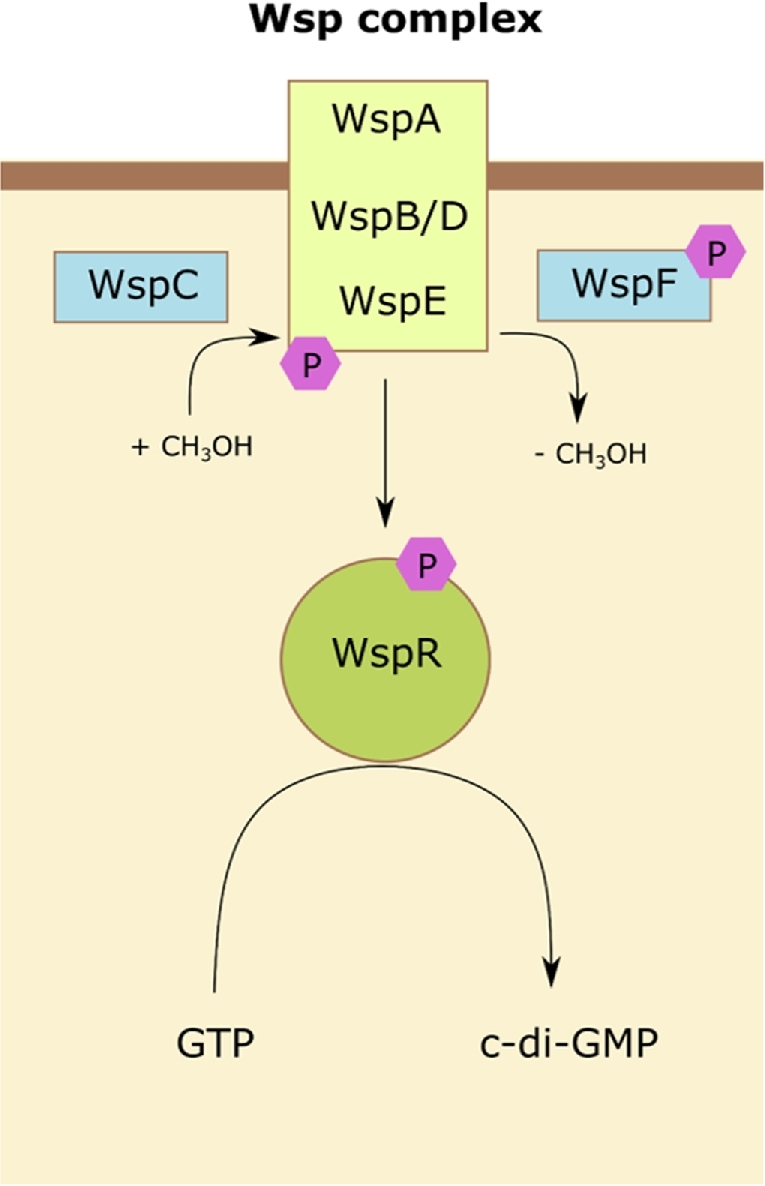
The Wsp pathway regulates c-di-GMP levels in response to environmental cues. The Wsp signalling complex consists of the receptor, WspA, scaffold proteins, WspB and WspD, and kinase, WspE. Upon activation by WspA, WspE phosphorylates WspR switching on its DGC activity resulting in the production of c-di-GMP. The methyltransferase, WspC, and methylesterase, WspF, regulate the activation of WspA.

Approximately 40 proteins are thought to be involved in *P. aeruginosa* c-di-GMP metabolism (Kuchma *et al.*^2007^). When *Pseudomonas aeruginosa* makes contact with a surface, the membrane-bound receptor, WspA, becomes activated and triggers c-di-GMP production by the DGC, WspR (Fig. 3) (Hickman, Trifrea and Harwood 2005; Blanka *et al.*^2015^). The c-di-GMP produced by WspR downregulates the motility apparatus (flagella), thereby forcing the bacteria into a sessile growth mode. Concomitant with this, the production of matrix components including exopolysaccharides, adhesive pili, eDNA and adhesins becomes upregulated by increased c-di-GMP levels (Römling, Galperin and Gomelsky 2013). Mutants such as those deficient in WspF function (a methylesterase that normally removes methyl groups from WspA, thereby decreasing its signalling activity to WspR) frequently arise in populations within the CF lung. In these mutants, WspA becomes constitutively activated, resulting in increased WspR phosphorylation and consequently, increased c-di-GMP production. This, in turn, upregulates expression of the *pel* and *psl* operons (Hickman, Trifrea and Harwood 2005; Blanka *et al.*^2015^).

SadC is another DGC that promotes the production of Psl, Pel and alginate even more so than WspR, despite WspR producing more c-di-GMP. This demonstrates the importance of subcellular pools of c-di-GMP, over total intracellular levels, in the activation of target effectors. SadC is located on the inner membrane and activates c-di-GMP synthesis in response to sensing Psl polysaccharide. Interestingly, SadC signalling activity is significantly increased following oxygen limitation (Schmidt *et al.*^2016^; Zhu *et al.*^2016^). Pel production and biofilm formation is reduced in *sadC* mutants, whereas swarming motility is enhanced (Merritt *et al.*^2007^). Another set of *sad* genes include the *sadARS* three-component system composed of a sensor kinase (SadS) and two response regulators (SadA/R). SadS triggers the activation of SadA/R; the EAL domain of SadR has been associated with PDE activity and so may regulate intracellular levels of c-di-GMP. Not only do they promote biofilm formation but the *sadARS* genes are also important in downregulating the virulence-determining type three secretion system (T3SS) during biofilm formation to maintain a sessile lifestyle (Kuchma, Connolly and O’Toole 2005).

The YfiBNR signal transduction pathway also modulates intracellular levels of c-di-GMP and is important for the induction of biofilm formation in response to high osmolarity. YfiN is a membrane-associated DGC, whose activity is negatively regulated by interaction with the periplasmic protein, YfiR. In turn, the activity of YfiR is regulated through its ability to interact with an outer membrane/peptidoglycan-binding protein, YfiB. YfiB sequesters YfiR, thereby preventing it from inhibiting the DGC activity of YfiN, leading to increased c-di-GMP production (Malone *et al.*^2012^).

**Figure 4.**
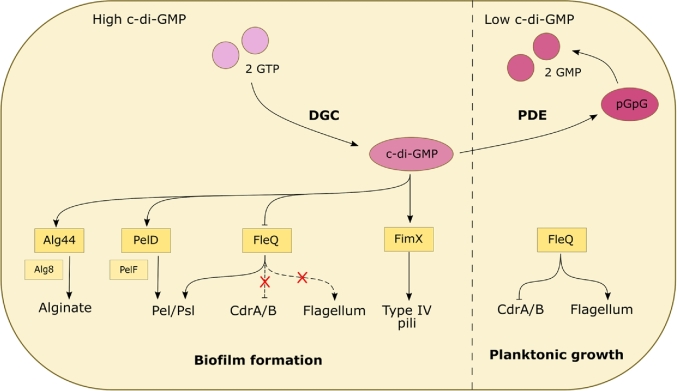
C-di-GMP is the main regulator of biofilm formation in *P. aeruginosa*. Diguanylate cyclases and phosphodiesterases regulate the intracellular pools of c-di-GMP. High intracellular concentrations of c-di-GMP have been found to promote the formation of biofilms and suppress planktonic growth. It does this by affecting a diverse range of effectors that control cell motility and the production of extracellular matrix components.

One of the main effectors of c-di-GMP is the transcriptional regulator FleQ which binds the ATPase FleN to promote the expression of flagellar biosynthesis genes. However, *fleQ* mutants also exhibit increased transcription of *pelA* and *pslA* genes, indicating that the c-di-GMP-dependent transcriptional upregulation of exopolysaccharides is mediated (at least, in part) through FleQ (Hickman and Harwood 2009). When c-di-GMP concentrations are high, the molecule binds to and induces major conformational changes in FleQ, which alters the affinity of this regulator protein for promoter sequences. In essence, c-di-GMP-bound FleQ no longer promotes the expression of flagellar synthesis genes, but instead, is converted to a transcriptional activator of the exopolysaccharide synthesis genes, promoting the biofilm lifestyle (Fig. 4) (Matsuyama *et al.*^2016^). In the absence of c-di-GMP, FleQ also represses transcription from the *cdrAB* promoter. However, in the c-di-GMP-bound form, FleQ dissociates from the *cdrAB* promoter, alleviating the repression and inducing *cdrAB* expression (Borlee *et al.*^2010^).

C-di-GMP also mediates the synthesis of alginate. Alg8 is an enzyme involved in the control of alginate polymerisation, and is regulated by a membrane-associated protein called Alg44. C-di-GMP-bound Alg44 activates Alg8 to synthesise alginate and target its transport across the cell envelope (Merighi *et al.*^2007^). It is thought that a similar mechanism upregulates Pel exopolysaccharide synthesis too. Upon binding c-di-GMP, the inner membrane protein, PelD, activates the Pel polymerase enzyme, PelF, thereby facilitating the export of Pel polysaccharide out of the cell (Lee *et al.*^2007^).

Another biofilm-associated process controlled by c-di-GMP is type IV pili (T4P) formation. These polar filaments are vital for twitching motility and contribute to the early stages of biofilm formation by promoting attachment to surfaces. FimX is a regulator of T4P which directly binds c-di-GMP, inducing a conformational change which activates its ability to positively regulate T4P assembly at the cell poles (Qi *et al.*^2012^). Because of this, deletions within the *fimX* locus results in poor biofilm formation (Navarro *et al.*^2009^).

C-di-GMP also inhibits a planktonic lifestyle by preventing the transcription of acute virulence factors. Vfr (virulence factor regulator) is a global virulence regulator that works in conjunction with its coregulator, cyclic adenosine monophosphate (cAMP), to induce the expression of genes associated with planktonic growth such as flagellum biosynthesis and both T2SS and T3SS. C-di-GMP interferes with this process by reducing the levels of cAMP but the mechanism by which this occurs remains unclear (Almblad *et al.*^2015^). Interestingly, in some *Pseudomonas* species c-di-GMP has been found to regulate secretion systems directly via the ATPase. C-di-GMP represses T3SS while promoting T6SS, consistent with the induction of biofilm formation by high intracellular c-di-GMP (Trampari *et al.*^2015^).

**Figure 5.**
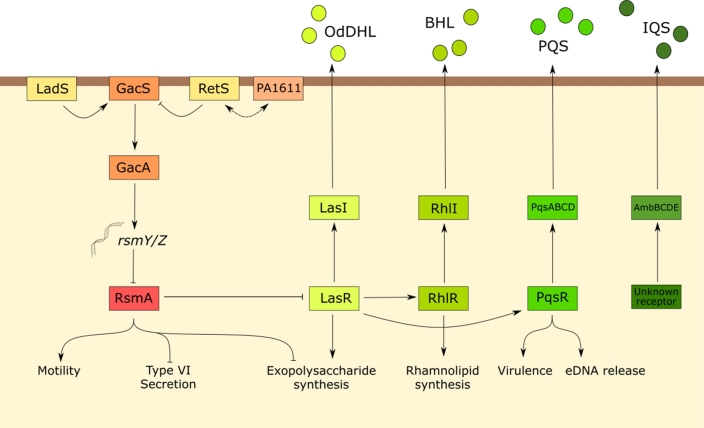
The Gac/Rsm pathway controls a diverse range of processes. In *P. aeruginosa*, RsmA promotes a planktonic lifestyle by upregulating motility apparatus and affecting the production of virulence factors by activating QS systems. Interestingly, QS is also implicated in promoting biofilm formation and plays a role in maintaining the biofilm matrix.

In addition to c-di-GMP, the Gac/Rsm pathway is also known to modulate biofilm formation through its control over exopolysaccharide production. This pathway is based on a two-component system in which the sensor histidine kinase, GacS, modulates the phosphorylation level of its cognate response regulator, GacA. The signalling activity of GacS is controlled by two sensor kinases, RetS and LadS, which work antagonistically to control GacS kinase activity. Phospho-GacA upregulates the transcription of two small RNAs, *rsmY* and *rsmZ*. Each of these RNA molecules can bind multiple copies of the small RNA-binding protein RsmA, thereby reducing the concentration of ‘free’ (unbound) RsmA in the cell (Moscoso *et al.*^2014^) (Fig. 5). A hybrid histidine kinase, encoded by PA1611, is structurally very similar to GacS and can interact with RetS to interfere with RetS-GacS activation. For this reason, the hybrid histidine kinase can induce the expression of genes controlled by RsmA (Chambonnier *et al.*^2016^).

Free-RsmA can function as a repressor by binding target mRNAs, promoting their degradation by RNases; for example, the T6SS is thought to be downregulated in this way (Frangipani *et al.*^2013^). However, the mechanism underpinning repression of the *pslA* transcript is more complex. RsmA binds to and stabilises a stem-loop structure in which the Shine-Dalgarno (SD) sequence of *pslA* is made unavailable through basepairing with an anti-SD sequence, preventing access by the ribosome and abolishing *psl* gene translation (Irie *et al.*^2010^).

RsmA can also function as an activator, and upregulates motility apparatus, including flagellar and pili-associated genes. RsmA also positively regulates T3SS and plays an important role in balancing virulence and planktonic growth with T6SS and biofilm formation (Moscoso *et al.*^2011^). The Gac/Rsm pathway influences production of quorum-sensing (QS) signals, as RsmA negatively regulates the *las* and *rhl* QS subsystems by binding to and inhibiting the expression of transcripts involved in QS regulation. Consequently, *gacA* mutants exhibit decreased QS and lowered production of QS-regulated virulence factors (Pérez-Martinez and Haas 2011).

The stimuli that activate RetS and LadS are still largely uncharacterised; however, recently calcium has been found to bind to the periplasmic domain (DISMED2) of LadS activating its kinase activity and ultimately promoting biofilm formation. The DISMED2 domain of LadS closely resembles some carbohydrate-binding modules; however, the presence of an additional helix inhibits this binding but allows for the binding of calcium instead (Broder, Jarger and Jenal 2016). RetS also contains a DISMED2 domain but so far the only known environmental stimuli that activate RetS comes from neighbouring lysed cells. It is thought that these lysing cells act as danger signals promoting RetS-dependent de-repression of the T6SS (LeRoux *et al.*^2015^). In addition, there are three two-component systems, BfiSR, BfmSR and MifSR, which become sequentially phosphorylated resulting in activation of GacS by BfiS. These two-component systems act independently of motility and exopolysaccharide production to control transition events from reversible attachment to late maturity during biofilm development (Petrova and Sauer 2009).

Besides c-di-GMP and Gac/Rsm pathways, QS has also been found to impact upon biofilm formation. *Pseudomonas aeruginosa* utilises four QS systems, the acyl-homoserine lactone (AHL)-based systems, *las* and *rhl*, the alkyquinolone system, *pqs*, and the recently identified integrated QS (IQS) system which responds to phosphate limitation. These interlink in a hierarchical manner with the *rhl* and *pqs* systems under the control of the *las* system (Jones *et al.*^1993^; Ochsner and Reiser 1995; Kirisits and Parsek 2006; Lee *et al.*^2012^). QS molecules are secreted into the environment and are taken up by neighbouring cells. As the bacterial population grows, accumulated QS signal molecules exceed a critical threshold concentration and trigger the coordinated expression of a wide range of genes, most noticeably virulence-associated genes (Arevalo-Ferro *et al.*^2003^; Davenport, Griffin and Welch 2015). Paradoxically, and despite increased virulence being associated with the planktonic lifestyle, studies have found that under some conditions, QS also enhances biofilm formation. The mechanism by which this occurs is not known but it is postulated to work through RpoS, the stationary-phase sigma factor, potentially through the upregulation of the *psl* operon (Diggle *et al.*^2003^; Irie *et al.*^2010^).

QS mutants often have changes in colony morphology indicating alterations to matrix structure. *LasI* mutants form flat colonies and produce very fragile pellicles in liquid culture reminiscent of *pelA* mutants. Consistent with this, the *pel* operon was found to be positively regulated by the *las* QS subsystem and also partially by *rhl* subsystem. LasR has also been found to bind directly to the *psl* promoter, although the significance of this association is currently not clear (Sakuragi and Kolter 2007). *rhl* QS is implicated in the maintenance of the biofilm by regulating rhamnolipid biosynthesis during macrocolony formation. Rhamnolipids influence cell–cell and cell–surface interactions to help maintain the channels that run throughout the biofilm supplying nutrients and removing metabolic waste. But again, the mechanism underpinning this maintenance remains unclear (Davey, Caiazza and O’Toole 2003). The *pqs* system however is thought to regulate the release of eDNA by inducing lysis in a subpopulation of cells within the biofilm. Low iron concentration stimulates the upregulation of *pqs* genes and the subsequent formation of eDNA. Typically, there is intense competition for available iron between host and bacterial cells of the CF lung and so this environment promotes biofilm development (Yang *et al.*^2007^; Häussler and Becker 2008).

QS signals have now been developed as biomarkers for biofilm formation. Clinical isolates have a characteristic ratio of *rhl*:*las* (acyl-HSL) signalling molecules. When grown in planktonic cultures, clinical isolates predominately produce *rhl* signal (BHL) whereas domesticated laboratory strains produce more *las* signal (OdDHL). However, when the laboratory strain, PAO1, was grown as a biofilm, the QS profile inverted and resembled more the acyl-HSL pattern observed in CF sputum samples. Using acyl-HSL profiles as biomarkers has since provided evidence that *P. aeruginosa* does in fact exist primarily as a biofilm in the CF lung (Singh *et al.*^2000^).

The aforementioned regulatory pathways do not work in isolation and are constantly feeding into one another to create a complex network that fine tunes the bacteria's response to environmental changes. Unfortunately, in most instances the environmental cues remain unknown but it is likely that the bacteria react to the high-stress environment of the lung in order to protect against dehydration, ROS and oxygen limitation (Mulcahy and Lewenza 2011).

## CONCLUDING REMARKS

*Pseudomonas aeruginosa* is one of the most highly studied and clinically significant organisms in relation to biofilm formation (Müsken *et al.*^2010^). It is now well documented that extracellular polysaccharides are vital for healthy and well-structured biofilms by contributing to stability, cell communication and antimicrobial evasion. Regulation of these exopolysaccharides, along with other matrix components, appears to be highly complex involving several interlinking signalling networks responding to different, and often undefined, environmental stimuli.

The presence of a heterologous population makes finding antibiotic treatment that is effective on the whole biofilm extremely difficult. For example, cap-forming and stalk-forming subpopulations will often display differential tolerance levels to antimicrobial compounds (Yang *et al.*^2007^). The tight colony structure of the biofilm also contributes to antimicrobial resistance and so it is becoming of increasing interest to directly target components of the biofilm matrix. Inhibition of biofilm development usually involves either preventing matrix synthesis or targeting its regulatory mechanisms. Exopolysaccharides have often been identified as attractive therapeutic targets for the disruption of biofilm formation, leaving the bacteria vulnerable to antimicrobial treatment. Anti-Psl antibodies can be obtained from CF patients recovering from *P. aeruginosa* infections showing that targeting Psl is important in the body's own attempts to disperse persistent biofilms (Baker *et al.*^2015^). Using polysaccharide lyases and digestive enzymes could also be used to improve the effectiveness of antibiotic killing. Preventative methods could involve targeting adhesins by preventing initial attachment and establishment of the biofilm. Targeting eDNA with DNase I has been found to effectively treat biofilms during the early stages of development. Similarly, disrupting eDNA production by treatment with iron salts has been found to induce dispersal of the biofilm (Wei and Ma 2013).

Here we have highlighted how c-di-GMP signalling, QS and the Gac/Rsm pathway converge to coordinate the expression of exopolysaccharide production and inversely downregulate the expression of planktonic-associated phenotypes. Understanding the regulatory pathways that underpin the synthesis of matrix components means that more and more regulators can be targeted to prevent biofilm development, not just in CF infections but also in burn wounds and ventilator-associated diseases. The regulatory systems mentioned in this minireview are only the tip of the iceberg for biofilm regulation and so research must continue to uncover the secrets behind this resilient bacterial way of life.

## FUNDING

EM is supported by a Doctoral Training Grant from the MRC. Work in the laboratory of MW is supported by the BBSRC, NC3Rs and the Rosetrees Trust.

***Conflict of interest.***None declared.
